# Reaching unreachables: Obstacles and successes of microbial cultivation and their reasons

**DOI:** 10.3389/fmicb.2023.1089630

**Published:** 2023-03-07

**Authors:** Gabriela Kapinusova, Marco A. Lopez Marin, Ondrej Uhlik

**Affiliations:** Department of Biochemistry and Microbiology, Faculty of Food and Biochemical Technology, University of Chemistry and Technology, Prague, Czechia

**Keywords:** environmental microbiome, microbial ecology, dormancy, VBNC, growth factors, cultivation techniques, improved cultivation, difficult-to-culture microorganisms

## Abstract

In terms of the number and diversity of living units, the prokaryotic empire is the most represented form of life on Earth, and yet it is still to a significant degree shrouded in darkness. This microbial “dark matter” hides a great deal of potential in terms of phylogenetically or metabolically diverse microorganisms, and thus it is important to acquire them in pure culture. However, do we know what microorganisms really need for their growth, and what the obstacles are to the cultivation of previously unidentified taxa? Here we review common and sometimes unexpected requirements of environmental microorganisms, especially soil-harbored bacteria, needed for their replication and cultivation. These requirements include resuscitation stimuli, physical and chemical factors aiding cultivation, growth factors, and co-cultivation in a laboratory and natural microbial neighborhood.

## Introduction

The planet we know today is largely the result of the microbial activity in the biosphere. Earth’s smallest and simplest organisms created the conditions for the development of the vast number of life forms we all know. The microscopic world is even vaster, and its diversity is stunning, but it is very difficult to reach. Even though its existence has been acknowledged for several centuries, it has been very challenging to study its roles. A crucial advance in the study of “the unreachables” arose in the days of Robert Koch at the end of the 19th century. He established a causative relationship between a microbe and its impact on a host (disease). Koch’s postulates demanded the presence of a microorganism in pure culture, isolated from the host, to confirm the link between the pathogen and the disease. From this point on, microbes were no longer considered scientific curiosities, but rather modelers of our bodies and Earth’s ecosystems (Turnbaugh et al., 2007; Graham et al., 2016; Gilbert et al., 2018). Much more efforts have been taken over the following decades to study microorganisms: these progressed from the description of and fight against the most critical human and plant pathogens, which dramatically improved our quality of life, to the later investigations on the community composition of different environments, the most advanced of which used marker gene or metagenome sequencing (Lane et al., 1985; Lynch et al., 2012). In recent years, sequencing technologies have addressed many environmental and human health-associated issues, such as the analysis of microbial responses to contamination (Hemme et al., 2010), the discovery of novel taxa to be used for bioremediation, the discovery of novel producers of antibiotics (Ling et al., 2015), or revealing the co-occurrence of antibiotic resistance genes in different environments (Li et al., 2015), to name a few.

Microorganisms live in virtually any environment, including those considered extreme due to their high temperature, pH, salinity, or concentration of pollutants (Mirzaie et al., 2015; Mehetre et al., 2018; Panda et al., 2018; Power et al., 2018; Maza et al., 2019). The physiological and biochemical potential of microbes living within these extreme environments is enormous. Thriving at the limits of life, extremophilic and extremotolerant microorganisms can provide enzymes such as the widely used Taq polymerase isolated from *Thermus aquaticus* (Brock, 1967; Brock and Freeze, 1969); or uncommon metabolites, such as previously unknown lipids (Schneider et al., 2019), unusual polyunsaturated fatty acids (Řezanka et al., 2019), antioxidants, pigments (Asker et al., 2012), bioactive natural compounds and other secondary metabolites with a wide range of applications (Lewis et al., 2010; Manivasagan et al., 2014). Microbes can also offer improved bioremediation possibilities (Pascoal et al., 2020), can assimilate unusual substrates (Xu R. et al., 2018) including toxic compounds, or resist and detoxify several antibiotics (Rettedal et al., 2014; McLain et al., 2016).

**Figure 1:**
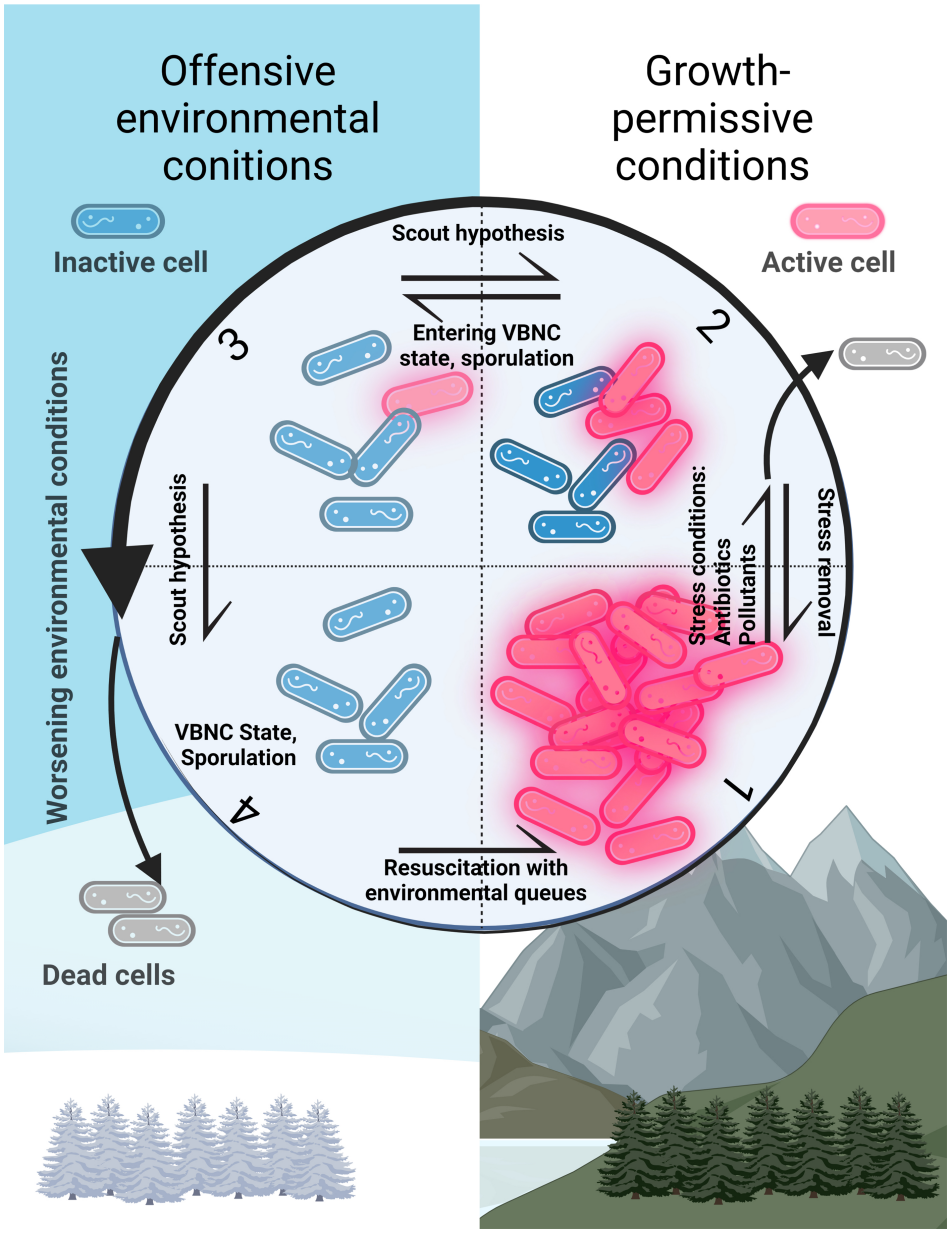
Different activity states of cells in the environment. 1: Active community or population. 2: In the presence of substances such as pollutants and antibiotics, a portion of the population dies and some cells can persist. These latter cells can then divide again when the substance is removed. 3: Cells in the viable but non-culturable state (VBNC). If a cell stochastically awakens in growth-permissive conditions (section number 2), the population starts replicating. If not, the cells die off (section number 4). 4: Cells in the VBNC state. Cells can resuscitate if environmental conditions become growth-permissive again (section number 1). This represents the resuscitation mediated by present environmental queues. Created with BioRender.com.

In order to fully describe these microorganisms and reveal their vast potential, it is necessary to obtain them in pure culture. Moreover, cultivation provides context to the metagenomic data (Nichols, 2007) and helps us verify metagenome-based conclusions on microbial interactions (microbe-microbe, microbe-plant, microbe-environment). However, bringing environmental microbes to pure culture under standard laboratory conditions has proven to be a very challenging task. Cultivation can be labor-intensive, tiring, time-consuming, and may not ensure success; but it can be rewarding if all the factors required for microbial growth are included (Figure 1). Here we discuss some generalities that elucidate the phenomenon of unculturability, with special attention paid to soil, being a habitat that harbors the greatest diversity of microorganisms, to build a foundation upon which to review some of the recent strategies to better reach “the unreachables.”

## Why do you not grow?

If it is alive, no microorganism is unreachable: we just do not know how to recreate their natural environment in order to obtain a pure culture (Watve et al., 2000; Stewart, 2012). With this in mind, the key step toward successful cultivation would be to replicate essential aspects of the microorganism’s natural existence as thoroughly as possible (Figure 1). Some of the environmental variables are easily discovered and can be readily incorporated into cultivation methodologies, but many other factors that influence growth are much more obscure, and including them in cultivation strategies is not as straightforward.

The environment in which microorganisms exist is usually different from the one we create for them in the laboratory. Microorganisms live under what Koch (1971) called a “feast and famine existence.” As a consequence, the growth dynamic observed under nutrient-rich laboratory conditions does not necessarily exist in nature, where environmental changes are common and poor nutritional conditions need to be withstood for longer periods of time (Koch, 2001; Pinto et al., 2015). Microorganisms can be categorized by their resource intake characteristics either as oligotrophs or copiotrophs (Meyer, 1994; Fierer et al., 2010). The main distinguishing parameters between these categories, as Ho et al. (2017) states, are their growth kinetics, substrate affinity, and efficiency at substrate utilization. Copiotrophs have higher Michaelis–Menten kinetics and maximal growth rate. Conversely, oligotrophs are slow-growing but have higher substrate utilization efficiency, and thus higher biomass yields per substrate molecule utilized. Oligotrophs thrive in environments with low nutrient flows, but not in substrate-rich/diverse environments. Copiotrophs, on the other hand, can utilize highly concentrated substrates rapidly and react promptly to substrate changes; they nevertheless lack the necessary regulatory mechanism of starvation, and are thus generally unable to grow in nutrient-poor sites (Ho et al., 2017).

The proportion of copiotrophs to oligotrophs in the environment, as well as under laboratory conditions, is governed by a dynamic process called succession (Fierer et al., 2010). Microbial communities change over time after they colonize a certain environment. For heterotrophic bacteria, organic carbon can be constantly supplied, i.e., exogenous succession, or present all at once at the initial colonization point, i.e., endogenous succession (Fierer et al., 2010). In the initial stage of endogenous succession, when nutrients are plentiful, copiotrophs are more abundant in the community; oligotrophs become dominant when highly concentrated substrates are depleted (Song et al., 2016). Both the changing environmental conditions in nature and an inappropriate choice of growth conditions in the laboratory hinder the ability of microorganisms to replicate and could thus render them dormant and seemingly unculturable.

## Do not wake up until it is beautiful outside

The low number of microbes cultivated in the laboratory compared with the total number of microorganisms observed under the microscope hinted at the existence of other states in which microorganisms may exist in nature, apart from being alive (replicating) or “dead” (non-replicating). This discrepancy, known as the “great plate count anomaly,” is a large difference, by several orders of magnitude, between the viable plate counts and the total direct microscopic counts (Staley and Konopka, 1985). This phenomenon reveals our failure to isolate all cells from a particular environment in pure cultures. Just as cells wait in a quiescent state for environmental conditions to be favorable again and start replicating (Kaprelyants et al., 1994), they can be waiting for these optimal conditions when deposited in the laboratory environment. Grandly said, microbes can be unreachable because they are “sleeping” (Xu et al., 1982).

The term “sleeping cells” encompasses several dormancy or quiescence phenomena that can cause unculturability under laboratory conditions. Dormancy is “any rest period or reversible interruption of the phenotypic development of an organism” (Sussman and Halvorson, 1966), or simply a state of metabolic inactivity as defined by Kell et al. (1998): cells exhibit negligible metabolic activity but can later transit to a growing state. This inactivity can be caused by the advent of unfavorable conditions, for example, the famine period in the dual feast-famine existence. Several dormancy phenomena have been identified, which suggests the existence of a “dormancy continuum,” where some states of dormancy can be deeper than others (Ayrapetyan et al., 2015). The most well-known state of dormancy is sporulation (Morrison and Rettger, 1930; Keep et al., 2006), in which some bacterial and fungal cells form spores as a survival strategy and outlast deleterious conditions. Spores then germinate when environmental conditions become favorable again.

Another dormancy-related phenomenon is that of “persistent cells,” first coined by Bigger (1944). This phenotype was already described in a study by Hobby et al. (1942), who observed that after exposing an infection-causing community to penicillin, 1% of the cells persisted. Persistent cells are non-growing phenotypic variants, completely dormant cells or cells inactivating genes selectively, frequently occurring in bacterial and fungal biofilms as small subpopulations (Harriott, 2019). They usually appear during the stationary phase or rarely in the exponential phase, and exhibit high tolerance to antibiotics (Wood et al., 2013). They avoid the antibiotic’s effects without undergoing genetic changes, so they play a significant role in population survival and biofilm re-creation (Lewis, 2010). In environmental biofilms, they create a subpopulation that supports biofilm survival against stress conditions such as starvation or other factors causing dormancy (Balaban, 2011; Carvalho et al., 2018).

Another common dormancy phenomenon is the viable but non-culturable (VBNC) state, believed to be widespread throughout gram-negative bacteria (Giagnoni et al., 2018). VBNC is a survival strategy that is similar to sporulation but present in non-sporulating cells (Mukamolova et al., 2003). It can be triggered by deleterious environmental changes, such as oxygen or substrate concentration changes or pH changes (Du et al., 2007). The inoculation of cells from their environment into artificial media can potentially trigger such a state. For example, when cultivating oligotrophs, the usage of a nutrient-rich medium can lead to cellular death; this may be a result of a depletion of energy for balanced growth or by osmotic shock caused by the sudden intake of non-metabolic complex substrates (Ho et al., 2017). In the VBNC state, cells do not replicate but remain viable after being exposed to stressful conditions (Xu et al., 1982). VBNC cells are also different from metabolically active and dividing cells, since they perform respiration and gene expression at low rates (del Mar et al., 2000; Shleeva et al., 2004; Li et al., 2014). They are also able to change their adhesion properties and virulence potential (Rahman et al., 1994; Du et al., 2007). Furthermore, their lower metabolic rate, strengthened cell wall and higher peptidoglycan cross-linking confer them better physical and chemical resistance, as opposed to normally dividing cells (Signoretto et al., 2000). When activity rates are reduced, VBNC cells also reduce their size (Biosca et al., 1996), increase their surface-to-volume ratio (Li et al., 2014), and, as a consequence, their nutrient intake increases (Baker et al., 1983). This size reduction was observed in *Burkholderia pseudomallei* and *Vibrio cholerae* cells when changing from rods during exponential growth to cocci in the VBNC state (Inglis and Sagripanti, 2006; Senoh et al., 2010).

**Figure 2:**
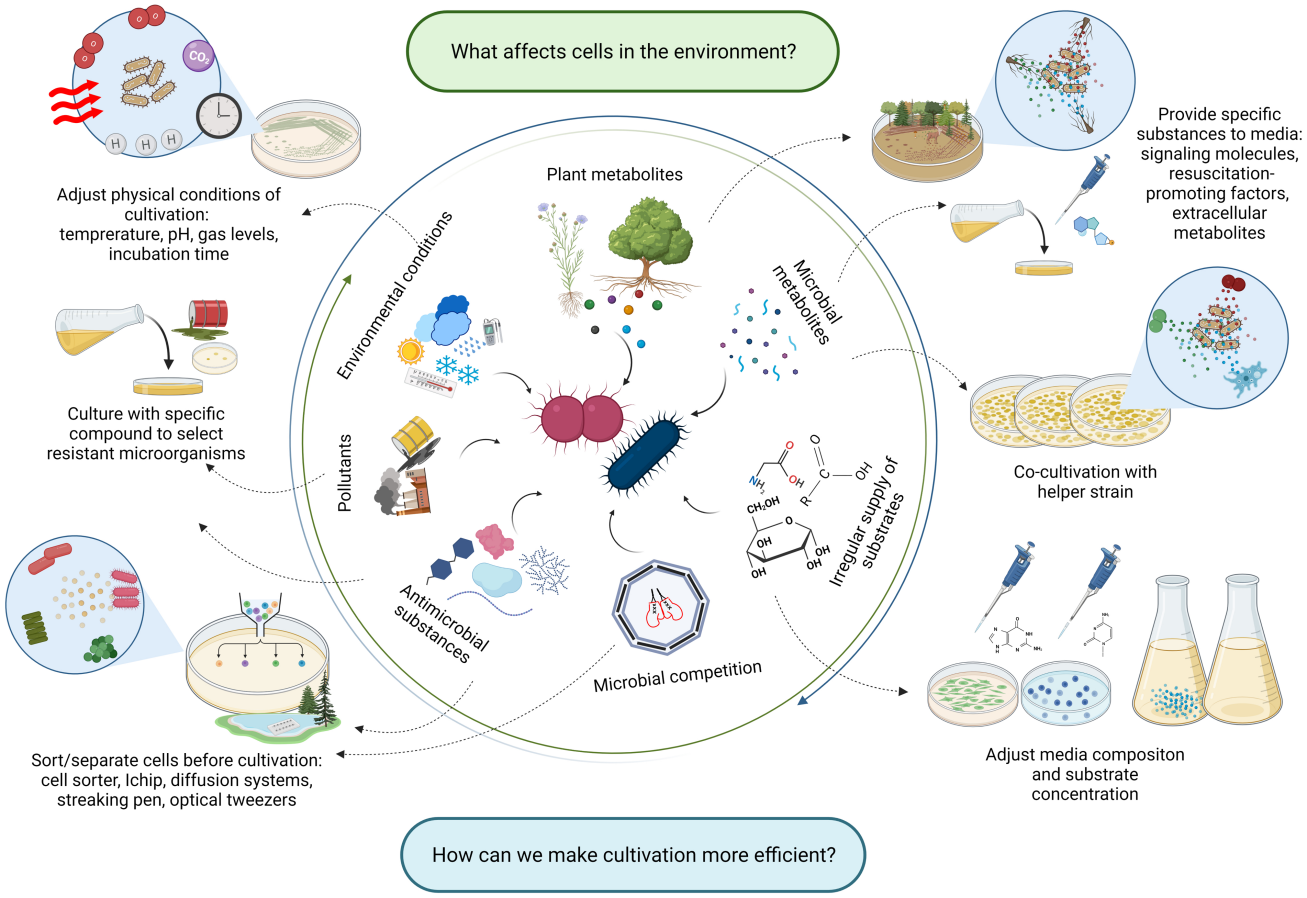
The list of factors affecting microorganisms in their environment (inner circle), and strategic approaches reflecting these factors in the cultivation (outer circle). Created with BioRender.com.

Dormancy can be one of the many reasons for unculturability, which biases the picture of the community observed *via* culturing methods. Fortunately, dormant cells are not totally unculturable but can be more challenging to culture because not only must their growth conditions be elucidated but also their resuscitation mechanisms. Two different mechanisms are thought to resuscitate microorganisms from dormant stages: either they depend on some environmental queue to do so or they do not, the latter situation being called the scout hypothesis (Epstein, 2009; Buerger et al., 2012). This stochastic reactivation of growth is the consequence of phenotypic variation within the dormant population (Sturm and Dworkin, 2015), which resembles the idea of the “dormancy continuum” previously mentioned. In both cases, knowing which factors are present in the environments where microorganisms dwell can teach us what is necessary for their effective culturing in the laboratory (Figure 2). If they wake up stochastically, they still need environment-resembling conditions where they can thrive after awakening. If they need environmental stimuli, then these would need to be included in *in vitro* cultivations for microorganisms to resuscitate and grow (Figure 2).

The stimuli needed to resuscitate microorganisms from dormancy include physical and chemical stimuli, which can be provided by the environment or by organisms to which yet-unculturable microbes are associated (Zhang et al., 2021). In this sense, the conditions needed to support growth in the laboratory medium can overlap with those to resuscitate microbes from dormancy but both phenomena correspond to different physiological processes, namely the exit from a reduced metabolic existence, after which comes the ability to replicate. Zhang et al. (2021) reviewed the factors that play a role in the resuscitation of VBNC organisms such as the addition of metabolites to minimize oxidative stress, quorum sensing autoinducers or temperature changes. In the present manuscript, the focus will be on those factors aiding the growth of microorganisms in the laboratory environment and what cultivation implies for modern microbiology.

## A helping hand from the environment – Physical and chemical factors

Temperature, pH, osmotic pressure, and oxygen and nutrient concentrations are ever-changing factors in the environment (Puspita et al., 2012). These changing conditions are stress factors that shape the composition of microbial communities as well as the environments in which they live. Soil pH has a major impact since it influences soil chemistry, including the availability of organic matter, redox conditions, and oxygen availability (Anderson et al., 2018). Energy-yielding metabolisms such as microbial respiration (Jin and Kirk, 2018) and the hydroxylated lipid membrane composition (Wang et al., 2016) also respond strongly to pH changes. The impact of pH on soil chemistry even shapes the assembly of microbial communities on a global scale (Feng et al., 2014; Tripathi et al., 2018). In this regard, according to a cross-continental phylogenetic survey of over eighty soils representing a wide range of ecosystems, soil pH was significantly correlated with the overall bacterial community composition (Lauber et al., 2009). The pH has been shown to significantly influence the community structure of other environments such as lakes (Ren et al., 2015), permafrost (Ren et al., 2018), and animal microbiomes (Sylvain et al., 2016). Even small changes in this variable can thwart growth on an artificial medium since some microorganisms have a very narrow zone of pH tolerance (Rousk et al., 2010). Adamberg et al. (2003) used a pH-auxostat to study the growth rate decrease of different lactic acid bacterial strains. A pH decrease from 6 to 4.3 was enough to slow down the bacterial growth rate, and ATP production was also lowered. However, microbial growth is not only affected by drastic changes in pH disabling microbial growth, but also by suboptimal pH, at which cell growth is detectable but the growth rate is significantly decreased, as was shown in the cultivation study of *Bacillus termoamylovorans* when pH changes by ~1.5 from the optimal pH for its growth caused a significant reduction in the growth rate and thus caused a reduction in energy yield per glucose molecule consumed (Combet-Blanc et al., 1995). However, there are cases when the microbes themselves, intentionally and unintentionally, are able to adjust the pH of their near environment, even by excreting basic metabolites or enzymes, and thus shape the microbial community and subsequently determine the interactions between individual species of the consortium (Ratzke and Gore, 2018).

Oxygen concentration also shapes the composition of entire microbial niches: whether it is oxygen-requiring algae, microaerophilic or facultatively anaerobic purple non-sulfur photoheterotrophs, anaerobic green-sulfur bacteria, or any chemotrophs, the development of individual subpopulations is impacted based on their relationship to oxygen. Not just the simple dichotomy of aerobic and anaerobic conditions is important, but also small, specific changes in oxygen concentration matter. For instance, *Coxiella burnetii*, the intracellular pathogenic agent of Q-fever, infects mammalian cells at a microaerobic concentration of O_2_ ~ 3%. Omsland et al. (2009) successfully cultivated an axenic culture of *Coxiella burnetii* on an improved acidified citrate cysteine medium under an oxygen tension of 2.5%–5%. Because of its ability to grow at lower oxygen levels, the hitherto uncultured *Coxiella burnetii* was able to utilize up to 17 different substrates and form visible colonies in the absence of host cells. Recently, *C. burnetii* was cultured in a modular hypoxic chamber that maintains the required O_2_ concentration (2.5%) without constant airflow, which greatly reduces the evaporation of the medium (Miller et al., 2020).

Oxygen concentration also induces oxidative stress caused by reactive oxygen species. Generally, the ideal oxygen conditions depend on oxidative stress sensitivity and the need for a reduced form of a nutrient (Vallejo Esquerra et al., 2017). Since reactive oxygen species often have a lethal effect on cells, it is desirable to reduce their concentration to a minimum. Oxidative stress during cultivation can be reduced by procedures such as autoclaving the agar and the phosphates separately (Tanaka et al., 2014; Kato et al., 2018, 2020) or by adding catalase or pyruvate to media (Bogosian et al., 2000; Tanaka et al., 2014).

Another decisive factor that enhances cultivation success is the choice of substrates and notably their concentration. Differing carbon concentrations create niches that are occupied by different bacteria (Eichorst et al., 2011; Wu et al., 2020). In environments prone to drastic environmental changes such as soil or water, selective pressure favors cells with a low metabolic cost existence (Mukamolova et al., 2003). Diluted, low-carbon media favor slow-growers and increase the overall diversity, thus increasing the chances of culturing unknown taxa. Low-carbon media have successfully increased the culturing of microorganisms coming from a wide range of environments, such as sea sponges (Karimi et al., 2019; Gutleben et al., 2020), aquatic environments (Imazaki and Kobori, 2010; Sun et al., 2019), or soils (Janssen et al., 2002; Molina-Menor et al., 2021). Aquatic environments offer the advantage of using the water directly from the source as part of the cultivation media. Applying this strategy, Kapinusova et al. (2022) isolated over 100 bacterial species, including several novel species of *Alphaproteobacteria*, *Betaproteobacteria*, *Flavobacteriia,* and even a member of a novel genus of *Thermoleophilia* (Kapinusova et al., 2022). A similar strategy combined with a prolonged incubation time was used for the culturomics of the world-renowed thermal springs of Karlovy Vary (Smrhova et al., 2022) and led to the acquisition of several thermotolerant strains of the *Bacillota* phylum and isolation of novel microorganisms of *Bacilli*, *Gammaproteobacteria*, and *Actinomycetia* classes. The dilution-to-extinction technique, based on the cultivation of soil oligotrophic microorganisms on media containing 100-fold diluted nutrients, resulted in the isolation of a wide spectrum of the most abundant soil representatives, and also of members of two previously undescribed actinobacterial lineages (Bartelme et al., 2020). The combination of the above-mentioned factors into one modified cultivation procedure, namely an adjusted N_2_/CO_2_ atmosphere (80:20), low substrate concentrations, the temperature corresponding to the original environment, etc., led to the successful isolation of members belonging to the OP5 phylum (Mori et al., 2008), first described by the 16S rRNA gene analysis in a hot spring in Yellowstone National Park (Hugenholtz et al., 1998).

Since many unreachables are slow-growers, prolonged incubation times can lead to their successful cultivation. Prolonged cultivations, usually coupled with culturing diluted cell suspensions, have proved to be useful in many studies (Eilers et al., 2001; Connon and Giovannoni, 2002; Rappé et al., 2002; Kakumanu and Williams, 2012; Adam et al., 2018; Bender et al., 2020). In a study by Davis et al. (2005), autochthonous soil cells, as well as non-native cells from constructed consortia, were counted on six different media at 7-day intervals. Cell counts increased even after 12 weeks of incubation. Another successful example of prolonged cultivation, and an important microbiological milestone, was the isolation of the previously uncultured archaeon *Candidatus* Prometheoarchaeum syntrophicum MK-D1 (Imachi et al., 2020). This extremely slow-growing Asgard archaeon, related to the *Lokiarchaeota*, was isolated from a 2,533 m deep-water sediment in the Nankai trough, Japan. Aiming to achieve deep-sea microbial cultivation, Imachi et al. (2020) set up a methane-fed-continuous bioreactor in which the enrichment cultivation ran for 2,000 days, resulting in the isolation of this archaeon from a symbiotic culture. The growth of some organisms from cold and oligotrophic environments, such as those isolated from Antarctica, can only be seen in culture after prolonged incubation times (Pulschen et al., 2017; Tahon and Willems, 2017). These organisms form very small colonies which often have to be observed under a microscope (Pulschen et al., 2017).

Longer incubations in a Petri dish or batch liquid medium can be problematic because the composition of the medium tends to change over time, either because of the action of the organism’s metabolism or other processes, such as water evaporation. Even though microbial species with apparently long cultivation times can have these incubations shortened upon subculturing (Buerger et al., 2012), their initial isolation from the natural environment could fail if they are cultured together with a faster-growing species. Slow-growing microorganisms can be disadvantaged mainly when microorganisms from complex consortia are attempted to be cultured together. Physically separating or sorting the microorganisms before their culturing is a helping strategy to overcome this problem and it is discussed further in the text.

Periodically varying conditions exist in nature, from the feast and famine cycles (Koch, 1971) to alternating oxic and anoxic periods (Dorofeev et al., 2019) and seasonality (Steiner et al., 2020), all of which can affect microbial communities. Besides culturing in continuous cultures (open systems) or batch cultures (closed systems), cyclic cultivation can be useful for microorganisms with a cyclic type of metabolism. This metabolism is divided into two phases: first, energy and carbon sources are accumulated, which are then used in the second phase to biosynthesize biomass (Dorofeev et al., 2014). Any of the above-mentioned culture parameters (e.g., temperature, oxygen, or substrate concentration) can be the cycling factor in the cultivation strategy (Dorofeev et al., 2014).

Some growth-influencing factors can be more enigmatic. One such factor is acoustic vibration, which is useful as a cultivation enhancement in several biotechnological studies (Bochu et al., 2003; Avhad and Rathod, 2015; Huang et al., 2017). By causing (i) cavitation and repairable damage in microbial cells, (ii) loosening of microbial aggregates in liquid cultures, and (iii) an increase in cell membrane permeability, ultrasonic low-intensity waves (∼20 kHz) can increase the substrate intake in microbial cells and subsequently enhance microbial proliferation (Huang et al., 2017, 2021), and thus can help in the cultivation of the unreachables.

Similarly, all the aforementioned culturing parameters can be combined in a high-throughput fashion to describe as much of the community composition as possible using cultivation, with each condition used being “a different aspect of the community’s picture.” This approach is referred to as culturomics (Greub, 2012). Bacteria obtained in culture are massively characterized using MALDI TOF-MS, or 16S rRNA gene sequencing (Strejcek et al., 2018; Nowrotek et al., 2019).

## A helping hand from the surroundings – Carrier particles

Many prokaryotes prefer to live attached to surfaces rather than in a dispersed, single-celled planktonic state (Mills, 2003; Flemming and Wingender, 2010; Hemkemeyer et al., 2018). In soils, different particle size fractions (PSFs) have a different impact on the concentration, chemical composition, and availability of organic matter (Christensen, 1992; Hemkemeyer et al., 2018). Organic matter is associated with fine-sized particles such as silt and clay; nevertheless, the sand fraction contains most of the free particulate organic matter (POM; Christensen, 2001), and therefore represents the fraction with the highest availability of substrates. The reported reduction in diversity among larger-sized fractions can be caused by low nutrient availability, protozoan grazing, and competition with fungi (Sessitsch et al., 2001). Hence, Hemkemeyer et al. (2018) observed the suitability of different PSFs and their associated POM to harbor microbial communities differing in their structure, functional potential, and sensitivity to environmental conditions. Genetic fingerprinting showed very strong preferences of the observed bacterial communities (up to 56% OTUs) for specific PSFs, while the archaeal populations did not exhibit significant preferences. Members of *Bacteroidota* and *Alphaproteobacteria* preferred the sand-sized fraction with POM, while *Actinomycetota* and *Betaproteobacteria* preferred fine silt, *Planctomycetales* clay, and *Gemmatimonadales* coarse silt (Hemkemeyer et al., 2018).

If cells prefer living in close contact with surfaces, it can result in it being difficult for them to grow in liquid media. Surfaces composed of different materials such as glass, steel, or synthetic polymeric substances such as polyurethane foams can enhance the cultivation of biofilm-forming bacteria from different natural environments (Yasumoto-Hirose et al., 2006; Gich et al., 2012; Dellagnezze et al., 2016). Liquid media provide many advantages compared to solid media: they guarantee a homogenous distribution of nutrients and oxygen, while also facilitating the manipulation of cultures. Aiming to combine the benefits of liquid media while meeting the requirements of microorganisms that live attached to surfaces, liquid media can be improved by adding a small amount of gelling agents such as gellan gum, xanthan gum, or carrageenan (Das et al., 2015), glass beads (Nguyen et al., 2005; Droce et al., 2013), or sand (Suman et al., 2019). Adding these supplementary solid agents can help the microorganisms to attach to the surface but still live and divide in the liquid or semiliquid medium.

## A helping hand from your neighbors – Growth factors

Trace elements from the environment, apart from the carbon source, are necessary to guarantee growth *in vitro*. To give a simple example, genera of the slow-growing *Acidobacteriota* living in manganese-enriched environments benefit from the addition of this element into their growth medium (Costa et al., 2020). Complex matrices, such as soil, harbor many phylogenetically diverse microorganisms (Bahram et al., 2018) that not only participate in important biogeochemical cycles (Louca et al., 2019), but also create conditions that enable the growth of other microorganisms by sharing metabolites and essential growth substances (Schink, 2002). These molecules include those that play a role in quorum sensing, biofilm community cooperation, or in the mutualism between plants and plant-growth promoting organisms (Jacoby et al., 2017), such as rhizobacteria and endophytes (Papik et al., 2020). If a metabolite is available in the environment, microorganisms can lose the metabolic capability of producing it and thus become metabolically dependent on their neighborhood (Pande and Kost, 2017). The absence of neighbors in pure culture, and consequently the absence of the necessary metabolites, is then one of the reasons behind unculturability (Pande and Kost, 2017). Bacteria living in certain environments, such as endophytes, benefit from the use of highly specialized growth medium containing the environment’s original metabolites (Gerna et al., 2022).

With the above said, some bacteria can only grow in a pure medium when in co-culture with another community member, also called a helper strain, which can be a phylogenetically different bacterium or even a different organism such as an amoeba (Boilattabi et al., 2021). Co-culturing can be achieved either by direct culturing of the helper strain together with the bacterium of interest or by using spent supernatants as a proxy for the helper strain (Stewart, 2012). Spent supernatants are the media where the helper strain grew, so the supernatants contain the metabolites that are potentially essential for other members of the community. Microbes can also be cultured together with the host from their natural environments (Knobloch et al., 2019; Lopez Marin et al., 2021). High-throughput co-culture is also now possible with devices such as microscale microbial incubators (Ge et al., 2016), micro-petri dishes (Ingham et al., 2007), microfluidic devices (Frimat et al., 2011; Burmeister et al., 2019), or agarose-based microwell chips (Zhang et al., 2019), where hundreds of single cells can grow in parallel in individual compartments, sharing metabolites and necessary substances for growth. The latter approach has proved very helpful in culturing bacteria directly related to human health, such as antibiotic-resistant pathogens from the human gut (Versluis et al., 2019).

Metabolites from associated bacteria can provide nutrients or trigger other stimuli necessary for growth. As was previously mentioned, when water and nutrients are on the wane and the surrounding conditions are unfavorable, some cells can enter dormancy. Dormant cells can be resuscitated by different resuscitation stimuli (Pinto et al., 2015). There can be many sources of such stimuli, but they often include substances such as amino acids and peptides (Nichols et al., 2008; Pinto et al., 2011), metabolites such as N-acyl homoserine lactones (Batchelor et al., 1997), or resuscitation promoting factors (Mukamolova et al., 2006; Pinto et al., 2013; Lopez Marin et al., 2021). For example, in a study by Bruns et al. (2002), the signaling molecules cAMP and N-(butyryl)-DL-homoserine lactone (BHL) increased total bacterial counts in highly diluted inocula from aquatic environments by several orders of magnitude. Thanks to this effort, the previously uncultured bacterial clone G100, *Citreicella manganoxidans*, belonging to the *Rhodobacteraceae* family, was cultured (Bruns et al., 2002; Wirth and Whitman, 2018). Less ambitious but still hopeful results were provided by the follow-up studies of Bruns, where the addition of cAMP led to a 10% increase in MPN values (Bruns et al., 2003). Yet, in several studies where signaling compounds were used for increasing cultivation yields, the influence of cAMP on culturability has been disproven (Pernthaler et al., 2003; Sangwan et al., 2005).

The resuscitation promoting factor (Rpf) produced by *Micrococcus luteus* promotes bacterial resuscitation and growth in the same producing organism (Mukamolova et al., 2006), but can influence taxa distributed along several other phyla, such as *Pseudomonadota* and *Bacteroidota* (Su et al., 2018; Lopez Marin et al., 2021; Su et al., 2021). This small protein (16–17 kDa) with a lysozyme-like structure (Cohen-Gonsaud et al., 2005) promotes bacterial cell growth even at picomolar concentrations (Mukamolova et al., 1998; Sexton et al., 2015). Rpf-like encoding genes are distributed among other prokaryotic genomes, especially in G + C rich gram-positive *Actinomycetota* (Nikitushkin et al., 2016), but Rpf-like proteins extend to other bacterial phyla, such as *Bacillota* (Shah and Dworkin, 2010) and *Pseudomonadota* (Li et al., 2020). The addition of Rpf during cultivation has resulted in the isolation of novel bacteria, such as organisms of the genera *Rhodococcus* and *Arthrobacter*, or of the family *Alcaligenaceae* (Su et al., 2013, 2015, 2018, 2021). Lopez Marin (Lopez Marin et al., 2021) isolated 51 novel bacterial species belonging mainly to the phyla *Actinomycetota*, *Pseudomonadota*, and *Bacteroidota* on reasoner’s 2A (R2A) agar and an agar made from the soil’s water-soluble fraction after supplementing *Micrococcus luteus* Rpf-containing supernatant to soils. Some of these species were members of novel genera, such as *Pedomonas mirosovicensis* of the family *Sphingosinicellaceae*, or *Solicola gregarius* of the family *Nocardioidaceae* (Lopez Marin et al., 2022, 2023). Spent supernatants containing growth factors have also aided the cultivation of *Chloroflexota* strains (Xian et al., 2020) or *Leucobacter*, the growth of which was supported through the action of zincmethylphyrins and coproporphyrins produced by *Sphingopyxis* sp. (Bhuiyan et al., 2016).

## Do you want to stay in your neighborhood?

The identification of specific substances promoting cell growth is not an easy task. To bypass the search for crucial growth factors, microorganisms can be co-cultured with growth-promoting microorganisms or can be cultivated *in situ* in the environments they come from Bollmann et al. (2007) and Remenár et al. (2015). *In situ* cultivation allows for the isolation of microorganisms that are more adapted to the original environment than those originating from the same habitat but obtained on standard agar media (Jung et al., 2016). Several innovative devices have been envisioned to deal with *in situ* cultivation. In an early attempt, Kaeberlein et al. (2002) developed a diffusion chamber that allowed the nutrients from the natural environment to migrate to the site where bacteria were inoculated. Seawater solidified with agar was sandwiched between two polycarbonate membranes, which allowed the flow of nutrients from the natural environment to the agar while at the same time isolating the inoculum from the natural environment (Kaeberlein et al., 2002). Diffusion chambers have since increased the diversity of culturable bacteria (Bollmann et al., 2007), including those that are difficult to culture, such as members of the phylum *Verrucomicrobiota* (Pascual et al., 2017) or bacteria highly resistant to heavy metals (Remenár et al., 2015).

A similar device to the diffusion chamber is the soil substrate membrane system (SSMS), which allows the growth of colonies over a membrane (made of materials such as polycarbonate), through which the nutrients and growth factors of the natural environment permeate and reach these colonies (Ferrari et al., 2005). Using the SSMS, Ferrari et al. (2005) isolated previously uncultured members of the genera *Aminomonas*, *Nocardia*, *Pseudomonas,* and *Enterobacter*. This membrane system has also been used to recover hydrocarbon-degrading bacteria from diesel-spiked polar soils (van Dorst et al., 2016) and was proven to recover rarer bacterial taxa from ice-free polar desert compared to conventional cultivation approaches (Pudasaini et al., 2017).

Later modifications of the diffusion chamber have been designed to culture microorganisms in the natural environment but using liquid media instead. One such early device was the hollow-fiber membrane chamber developed by Aoi et al. (2009). It is composed of hollow polyvinylidene tubes where microbes are inoculated and grown. The tubes are porous, so they allow the transport of molecules from the natural environment to the inside of the tube. In comparison with standard petri dish methods, the hollow-fiber membrane chamber technique yielded a higher ratio of novel phylotypes, mostly of *Pseudomonadota*, *Actinomycetota*, *Bacteroidota*, and *Spirochaetota*, and also resulted in an overall higher diversity of the recovered isolates (Aoi et al., 2009). Another liquid medium-based diffusion chamber is a bioreactor separated from the surrounding environment by a polycarbonate membrane (Chaudhary et al., 2019; Chaudhary and Kim, 2019). With this device, 35 previously uncultured bacteria belonging to the phyla *Pseudomonadota*, *Bacillota*, *Bacteroidota*, and *Actinomycetota* were isolated; the largest number of novel isolates was obtained when soil extract was used for the preparation of the medium (Chaudhary et al., 2019). Diffusion chambers have been manufactured in 3D printers, which increases their customization possibilities for their use in different applications (Wilson et al., 2019).

Diffusion chamber devices have been subject to further modifications. One such example is the so-called microbial trap, which consists of two semipermeable membranes with agar or gellan gum “sandwiched” between them (Gavrish et al., 2008). Filamentous *Actinomycetota* can access the medium from the outside through the semipermeable membranes. A similar trap was designed by Jung et al. (2013), with the difference that the trap’s access size can be modified. This latter trap has been used to culture various microorganisms from extreme environments, such as saline lakes (Jung et al., 2013) and hot springs (Jung et al., 2018). Yet another modification to the microbial trap uses sub-micrometer constrictions, where microorganisms compete to reach a chamber with nutrients going through a thin opening that allows only one bacterium to access and form a pure culture (Tandogan et al., 2014). Both groups of devices, diffusion chambers and microbial traps, have been shown to help reduce cultivation bias by culturing bacterial representatives which metagenomics approaches identified as the main representatives in a specific community (Pathak et al., 2020).

A successful high-throughput modification of the diffusion chamber technique is a system of multiple diffusion chambers called the isolation chip (iChip), first coined by Nichols et al. (2010). It consists of an assembly of three flat plates, a central one, and two symmetrical external plates. The external polyoxymethylene plates are provided with a set of 384 holes, since every chamber in the central plate is designed to capture, ideally, just one cell. The inoculated central plate is covered, as with Bollman’s device (Bollmann et al., 2007), with a standard polycarbonate membrane, which permits the flow of nutrients from the environment and at the same time keeps the cells inside the chambers. The external plates prevent the cells from migrating in and out, and also keep them literally trapped inside their chambers. This chip can then be placed in the natural environment to serve as a cultivation chamber *in situ* (Berdy et al., 2017). Among others, the Antarctic bacterium *Aequorivita* sp., possessing antimicrobial and anthelmintic activity, was isolated using the iChip system (Esposito et al., 2018; Liu et al., 2021). The iChip has also aided in the cultivation of antibiotic-producing bacteria, such as the bacterium *Eleftheria terrae*, which produces the antibiotic teixobactin (Ling et al., 2015).

Devices similar to the iChip have been used recently to culture fastidious bacteria. The diffusion sandwich system, a device based on the iChip, led to a successful culturing of *Pseudomonas soli* which can produce xantholysin congeners (Pascual et al., 2014) or the gellan gum-degrading bacterium *Luteolibacter gellanilyticus* (Pascual et al., 2017). Acuna (Acuna et al., 2020) used microwell chambers, devices similar to the iChip in design, to culture rhizobacterial populations. Rhizosphere microorganisms were also cultured *in situ* using the Rhizochip, an acrylic device with holes, in which microorganisms are randomly and not evenly inoculated, and placed into a plant rhizosphere (Gurusinghe et al., 2019). All these examples show that when the unreachables stay in their environments, we are more likely to reach them in cultures.

## Want to be sorted or isolated before cultivation?

Because of the enormous number of microorganisms awaiting cultivation, it is natural to assume that automation and high-throughput culturability will be more and more common. Organisms in a community can be individually sorted and cultured under a broad range of conditions. Among these sorting approaches are the preselection of cells by their size, shape, or by any other characteristic. This results in the division of the total microbial community into several subpopulations consisting of similar microorganisms. Such a separation requires equipment such as optical tweezers, flow cytometry coupled with sorting cell assays, or the integration of both methods (Tewari Kumar et al., 2020).

In 2002, Zengler and his team presented a method involving microdroplets of solidified agarose for encapsulating single bacterial cells. The encapsulated cells were then grown in a column with low nutrient media, and thus were able to grow “together but apart” (Zengler et al., 2002). This high-throughput cultivation method resulted in the growth and successful isolation of newly identified *Planctomycetales* and *Alphaproteobacteria* (Zengler et al., 2002). An advantage of this microdroplet cultivation is the broad range of environments to which the technology can be applied. Later, in 2005, Zengler presented an improved version of the method, Diversa’s high-throughput cultivation using microcapsules, by which it is possible to obtain more than 10,000 bacterial and fungal isolates from a matrix (Zengler et al., 2005). More recently, alginate microbeads have been successfully used for the high-throughput culturing of bacteria that usually resist cultivation such as *Verrucomicrobiota* and *Epsilonproteobacteria* (Ji et al., 2012), and also to cultivate anaerobes (Börner et al., 2013). Analogously to the co-culture strategy, bacteria grow in the presence of other members of the community, just separated from each other in individual capsules or drops. Encapsulated microorganisms can then be sorted, for example by using fluorescence-activated cell sorting, according to their phenotype of interest (Eun et al., 2011) or other distinguishing properties such as the presence or absence of growth in each droplet (Zang et al., 2013; Ota et al., 2019), their growth rate (Akselband et al., 2006; Ota et al., 2019), chemotactic motility (Dong et al., 2016), or their metabolic activity (Espina, 2020).

Focusing on slow-growing microorganisms after sorting can result in the cultivation of rare taxa (Watterson et al., 2020). Jian and coworkers developed a microbial microdroplet culture system, where cells are cultured in water-in-oil droplets placed in Teflon tubes. This system uses up to 200 droplets with a volume of 2 μL, in which microbes are cultured in a high-throughput fashion (Jian et al., 2020). The droplets can be manipulated to meet the needs of different experimental designs. Microbe-harboring beads or droplets (or, in general, sorted cells) can also be cultured in their natural environments, which can be achieved by encapsulating the beads inside an extra polysulfonate membrane to isolate the encapsulated cells from the environment (Ben-Dov et al., 2009) or, more recently, using devices such as the Microbe Domestication Pod (Alkayyali et al., 2021). The pod, which holds agarose microbeads containing encapsulated cells, is placed in the environment, allowing the encapsulated microorganisms to be cultured individually but guaranteeing cell-to-cell communication and the presence of important environmental necessities.

Sorted droplets can also be placed in microwell slides in order to facilitate downstream cultivation and analysis (Bai et al., 2014). The sorting of cells in compartments can also be exploited to research cell-to-cell interactions among encapsulated bacteria (Ohan et al., 2019) and biofilm formation or growth (Chang et al., 2015; Jin et al., 2018). The elucidated interactions can cast light upon each cell’s needs for growth, and thus on its effective cultivation. Devices such as the SlipChip, composed of two conjoined plates, allow the duplication of a microbial colony so that half of it can be further preserved or cultured, while the other half can be used for destructive analyses (Ma et al., 2014).

Another way to sort the unreachables is to separate them while growing on a petri dish. Cultures can be sprayed onto medium plates instead of being spread with a hockey stick. This procedure effectively compartmentalizes microorganisms in droplets, hence the aggregation of cells and interspecies competition, once they land on the medium, is significantly reduced (Huang et al., 2021). Gao and co-workers developed a microbe observation and cultivation array (MOCA) that allows the recovery of microbes on a small scale and does not require any complex equipment (Gao et al., 2013). MOCA involves a petri dish with arrays of oil-covered droplets of cells. The oil covering provides a separation between cells and thus enables the cultivation of multiple separated droplets of cells (Gao et al., 2013). Several marine microorganisms were isolated using this technique, including *Pseudoalteromonas* spp. and previously uncultured members of the genera *Shewanella* and *Colwellia* (Gao et al., 2013). Compared to conventional approaches, MOCA offers an easy system for compact, parallel cultivation and multiple variations of different media on a relatively small scale.

“Streaking pen” developed by Jiang and his group is a robust, high-throughput method based on a simple streaking and picking strategy to achieve single-cell cultivation on microfluidic streak plates. Using this technique, a previously unknown fluoranthene-degrading *Blastococcus* species was isolated (Jiang et al., 2016), and so were novel species of bacteria from a marine sediment (Xu B. et al., 2018; Hu et al., 2020). This method has also been used to culture termite-associated bacteria of the genera *Burkholderia*, *Micrococcus,* and *Dysgonomonas* (Zhou et al., 2019). In general, cell sorting enables the design of complex experiments using just a few plates, and thus represents a great experimental simplification that allows for a better examination of individual subpopulations and, as a result, increases the chances of culturing novel taxa.

## Let us seek information about the cultivation of the unreachables in the (meta)genome

Successful cultivation of just a few novel taxa while adding “vital” molecules to the media, trying different media and cultivation conditions, or the combination of all the above, is a lengthy and material-consuming way to find the requirements for microbial growth of specific taxa, given the vast diversity of the unreachables. Nowadays, the metagenome has become a promising source of information on cultivation needs, since it reveals “who is there and what their roles are” (Remenár et al., 2015; Nowrotek et al., 2019). In other words, why try dozens of media or condition combinations, when each cell’s growth requirements can be found in its genome?

As was mentioned earlier, a common phenomenon in a community is the loss of the ability to metabolize certain compounds if these are provided by other organisms (Pande and Kost, 2017). Such a gene loss can be ultimately seen in the genome (Carini et al., 2013). Reconstruction of the metabolic pathways through genomic information reveals the bacterium’s deficiencies or needs, which can be provided in the medium (Liu et al., 2022). For example, the nutritional requirements of *Pelagibacter ubique*, most likely the most abundant bacterium on Earth, were determined in part by its absence of genes for assimilatory sulfate reduction and its need for reduced sulfur compounds for growth (Carini et al., 2013). Karnachuk et al. (2020) isolated a thermophilic spirochete thanks to information from a metagenome-assembled genome which suggested the presence of 12 alpha-amylase hydrolases. This bacterium was then cultured using a medium composed mainly of starch (Karnachuk et al., 2020).

Metagenomic data can also be used to create co-occurrence network approaches based on network inference techniques in order to model the abundance or roles of specific community members in an environment (Faust and Raes, 2012). These relationships can be exploited in co-culture approaches, which can represent these relationships, e.g., by using spent media from other culturable bacteria in the community (Xian et al., 2020). Information contained in an RNA sequence (metatranscriptome) can be even more useful because it reflects the necessary genes being expressed in a given environment and time. For instance, the metatranscriptome of the leech *Hirudo verbana* was characterized, revealing the expression of genes coding for sulfated-mucin desulfatases and sialidases (Bomar et al., 2011). A medium with added mucin then allowed the cultivation of a *Rickenella*-like leech symbiont *in vitro* (Bomar et al., 2011).

A metagenome is a very complex collection of information, so discerning specific, individual genomes out of this mixture is often a difficult task, and techniques that provide a link between identity and function can help to discern which specific organisms carry which metabolic activity. One such technique is stable isotope probing (SIP), a method that links certain metabolic capabilities to individual community members. Upon probing with stable isotopes, the metagenome of these community members can be separated and sequenced to reveal their identity (Uhlik et al., 2013). SIP in tandem with metagenomics helped culture different bacteria with, for example, biodegradative functions. The bacterium *Polaromonas naphthalenivorans* was isolated in a pure culture after its role in the degradation of naphthalene was determined by SIP (Jeon et al., 2003, 2004). A similar approach was followed for isolating novel phenanthrene- and biphenyl-degrading *Ralstonia* populations (Li et al., 2019), novel isoprenedegrading bacteria belonging to different genera (Larke-Mejía et al., 2019), or hydrocarbon-degrading bacteria from the sea basin (Mishamandani et al., 2014; Gutierrez et al., 2015) or oil spills (Gutierrez et al., 2013). In these examples, the stable isotope-labeled, or “heavy” molecule used for the biodegradation analysis was also included in the cultivation efforts, but “heavy” genomes could also highlight other requirements that the degrading bacteria may need.

Finally, metabolic needs can be elucidated by single-cell genomics (Wurch et al., 2016), which can be boosted with the cell-sorting approaches described earlier. Single-cell genomic information has enabled metabolic reconstruction and aided the isolation of difficult-to-culture organisms such as symbiotic archaea (Wurch et al., 2016). Additionally, Cross et al. (2019), using single-cell genomic data, developed a method to capture specific microorganisms using antibody engineering. These antibodies are designed based on membrane-associated proteins, whose sequences can be found in the genome. The antibodies are labeled with a fluorescent dye, and thus the cells to which the antibody binds can be sorted by flow cytometry and cultivated in different media (Cross et al., 2019).

## Conclusion and future perspectives

**Figure 3:**
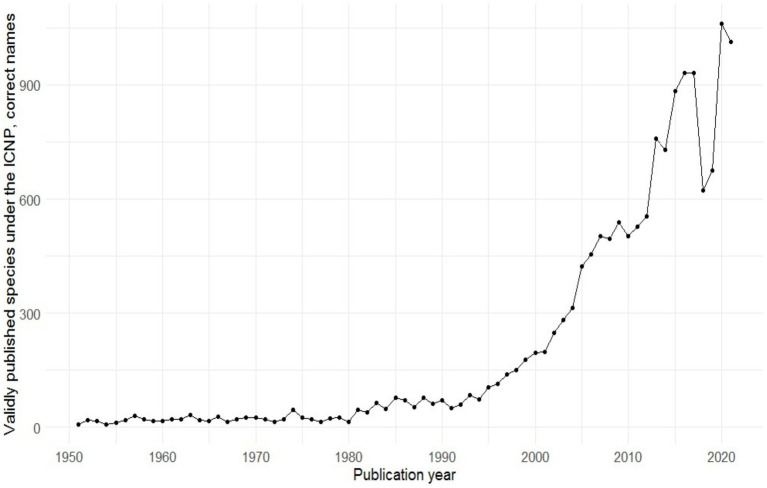
The number of validly published species within the last 70 years (Parte et al., 2020).

In recent years, a large number of microbes have been cultured employing the procedures discussed here in. Over the last two decades, in particular, a great deal of effort has been spent to improve culturing work, and many new taxa have been described; in fact, more bacteria have been cultured and described in the first 20 years of the 21st century than in all previous years of microbiological research combined (Figure 3; Parte et al., 2020). The high-throughput sequencing revolution that enabled the analysis of the metagenome has great potential to aid the cultivation progress. There is an unavoidable synergy between culture-independent and culture-dependent knowledge: as our knowledge of metagenomes increases, so does our knowledge of what microbes need to grow. The majority of Earth’s environments still harbor mainly hitherto uncultured microorganisms (Lloyd et al., 2018). Just as an ebb primarily uncovers areas close to the shore, and maybe never reveals the perpetually hidden abyss, so the phylogenetically distant cells, or “phylogenetically divergent non-cultured cells” as described by Lloyd et al. (2018), may remain undiscovered. These unreachables are the real “dark matter” of the microbial world and keep on shaping our planet right under everyone’s noses. But in theory, nothing is impossible to culture, and what we do not successfully culture today can be brought to culture tomorrow. Just like the Yellowstone National Park’s Obsidian Pool gave us a hint of the then so-called OP5 or OP10 phylum (Hugenholtz et al., 1998), whose members were isolated more than a decade later (Mori et al., 2008; Lee et al., 2011; Tamaki et al., 2011), other environments will reveal their secret inhabitants *via* culture-independent, omics-based approaches, after which culturing will be applied in search of their objectification. But not just simple, low-scale culturing; automatized, high-throughput culturomics will be needed. Sorting technologies such as those based on microfluidic systems could already be coupled with machine learning systems (Srikanth et al., 2021) so that growth needs can be elucidated and a high number of microorganisms can be cultured in the shortest time possible. This is the same as with many other big questions that still afflict us: it seems that machines and algorithms are coming to the rescue. So many microbes will be unreachable no more, and the time for this is already being reached.

As mentioned in the previous section, there may be an interest in culturing a specific organism from the environment and techniques have been proposed to tackle this challenge (Cross et al., 2019). Throughout the course of microbiological research, several taxa have been categorized as “most wanted” because of the important roles they play, such as in the human microbiome (Almeida et al., 2016) or other environments in the biosphere (Steele et al., 2011). At the same time, some microorganisms exist as obligate symbionts: their genomes have been reduced because of the loss of functional genes, and these lost functions can be guaranteed by the host (Moran and Bennett, 2014). Entire bacterial phyla such as the Candidate phyla radiation are thought to be composed mostly of symbionts (Castelle and Banfield, 2018). Should we force them to try to exist by themselves in a pure culture, despite their loss of basic structural features such as cell wall components and extremely small genomes (<200 Kbp) with maybe no possibility of growing away from their host, or should we better make more flexible regulations of what is required to propose new prokaryotic species? Cultivation is made difficult not only because of the intricacies needed for the growth of microorganisms in the laboratory but by placing unreal requirements for their study through culturing.

There are calls to reform the one species-one publication formula (Rosselló-Móra and Amann, 2015) and, due to the diversity of bacteria in the environment, it is not difficult to imagine that it may be impossible to describe all bacterial species using the polyphasic approach employed today for circumscribing new species, even if all microbes were culturable. Recent estimates suggest that the number of different bacterial taxa in the biosphere (established with a 16S rRNA gene similarity cutoff of 97%) is 2.2–4.3 million (Louca et al., 2019). New bacterial descriptions are also constrained by journal capabilities (Tamames and Rosselló-Móra, 2012). In order to give an identity to the mass of uncultured microorganisms, the availability of a pure culture is maybe not necessary anymore. High-quality genome sequences are being proposed as nomenclatural types (instead of viable anexic cultures in culture collections), and a new classification system, the SeqCode, is being developed to exist (at least temporarily) parallel to the International Code of Nomenclature of Prokaryotes (Hedlund et al., 2022; Whitman et al., 2022). The requirements of a pure axenic culture of the ICNP as the only type material possible for naming new microbial species has been criticized as self-limiting, hindering microbiological research and raising the costs associated with naming new taxa (Palmer et al., 2022). If the “dream of a phylogenetic system” was materialized upon the bases of genomics (Woese, 1992), the development of a reliable system based on genomics must be pursued and supported.

These recent developments in prokaryotic systematics will not negatively affect the importance of cultivation because microbiology is a science whose reach extends far beyond taxonomy and the basic knowledge of microbes. It is expected that, by 2024, the economic value of the global microbes and the microbial market will exceed USD 675.2 billion (Estevinho et al., 2020). These figures are reached by allocating organisms in high-value biotechnological industries which produce the goods previously mentioned in the introduction. The “dark matter of life” conceals not only the answer to “who is there,” but also “what are they doing.” This second question is still what may be most relevant contributing to the advancement of technology. The future of cultivation is one that begins with its strengths: the ability to select and culture microorganisms relevant to their functions and technological potential. But we must be open-minded enough to not limit our horizons with just apparent and obvious applications: a world of possibilities can be opened with each microorganism isolated and studied.

## Author contributions

GK and MLM contributed equally to the literature research and writing of the review, all under OU’s guidance and supervision. All authors approved the final version.

## Funding

Financial support is acknowledged of the Czech Science Foundation under grant no. 22-00132S and INTER-EXCELLENCE program of the Ministry of Education, Youth and Sports of the Czech Republic under grant no. LTAUSA19028.

## Conflict of interest

The authors declare that the research was conducted in the absence of any commercial or financial relationships that could be construed as a potential conflict of interest.

## Publisher’s note

All claims expressed in this article are solely those of the authors and do not necessarily represent those of their affiliated organizations, or those of the publisher, the editors and the reviewers. Any product that may be evaluated in this article, or claim that may be made by its manufacturer, is not guaranteed or endorsed by the publisher.

## Figures and Tables

**Table 1:** 

**bacterial persisters**	microorganisms that survive exposure to a given antibiotic/action that limits their cellular division, and have the capacity to replicate once it is removed (Zhang, 2014)
**community**	a multi-species group of organisms, living together in a shared environment and interacting with each other (Konopka, 2009)
**copiotrophs**	organisms adapted to utilize available resources promptly when available; usually associated with nutrient-rich environments (Koch, 2001). They have higher Michaelis–Menten kinetics and maximal growth rates
**co-culture**	biological systems (cultivation strategy) where two or more different microbial populations coexist with some degree of contact between them (Goers et al., 2014; Rosero-Chasoy et al., 2021)
**culturomics**	high-throughput, cultivation-dependent methods describing an environment’s microbial community (Lagier et al., 2018)
**dormancy**	a survival strategy characterized by a reduction in metabolic activity, usually undetectable under laboratory conditions (Dworkin and Shah, 2010)
**iChip**	an isolation chip consisting of a customizable set of chambers, where environmental cells are kept separately and subsequently cultivated *in situ* (Nichols et al., 2010)
**in situ incubation**	a cultivation method leading to the facilitated growth of cells that are difficult to cultivate *ex situ*, usually performed in the environment that the cell originated from Nichols et al. (2010) and Epstein (2013)
**metabolomics**	a metabolic profiling that links genotype and phenotype based on the targeting of small molecules (peptides, amino acids, nucleic acids, etc.; Zhang et al., 2012)
**metagenomics**	a study of the collective genomes of all microorganisms found in a given site/sample (Handelsman et al., 1998)
**metatranscriptomics**	a culture-independent microbial profiling based on their gene expression (Filiatrault, 2011)
**microbial succession**	change in the composition of microbial communities over time after the colonization of a new environment (Fierer et al., 2010)
**microbiome**	an entire habitat, including the microorganisms, their genomes, and the surrounding environmental conditions (Marchesi and Ravel, 2015)
**mixotrophs**	organisms relying on both heterotrophy and autotrophy (Crane and Grover, 2010)
**oligotrophs**	organisms capable of growing in low-nutrient environment/media (0.5–15 mg of C/L) and, conversely, unable to grow on substrate-rich media immediately after removal from their natural environment (Cho and Giovannoni, 2004)
**microbial population**	a collection of cells of one species living in the same environment and interacting with each other (Thompson, 2020; Behera et al., 2022)
**resuscitation-promoting factors**	factors enabling the cell division or resuscitation of dormant cells (Hett et al., 2008), usually referring to a protein/proteins of various gram-positive bacteria (*Mycobacterium* and *Micrococcus* genus; Mukamolova et al., 2002, 2006)
**single-cell-sorting**	a sorting device based on the compartmentalization of a heterogeneous mixture of particles/cells of different types (one or more), into different volumes (Seeger et al., 1991; Grover et al., 2001)
**viable but non-culturable (VBNC)**	a cellular survival strategy (Giagnoni et al., 2018) in which cells retain indicators of metabolic activity while being incapable of sustaining cellular division on media that normally support the growth of the microorganism (Rice et al., 2000)
